# A Quantitative Systems Approach Reveals Dynamic Control of tRNA Modifications during Cellular Stress

**DOI:** 10.1371/journal.pgen.1001247

**Published:** 2010-12-16

**Authors:** Clement T. Y. Chan, Madhu Dyavaiah, Michael S. DeMott, Koli Taghizadeh, Peter C. Dedon, Thomas J. Begley

**Affiliations:** 1 Department of Biological Engineering, Massachusetts Institute of Technology, Cambridge, Massachusetts, United States of America; 2 Department of Chemistry, Massachusetts Institute of Technology, Cambridge, Massachusetts, United States of America; 3 Department of Biomedical Sciences, Gen*NY*sis Center for Excellence in Cancer Genomics, University at Albany, State University of New York, Rensselaer, New York, United States of America; 4 Center for Environmental Health Sciences, Massachusetts Institute of Technology, Cambridge, Massachusetts, United States of America; University of Washington, United States of America

## Abstract

Decades of study have revealed more than 100 ribonucleoside structures incorporated as post-transcriptional modifications mainly in tRNA and rRNA, yet the larger functional dynamics of this conserved system are unclear. To this end, we developed a highly precise mass spectrometric method to quantify tRNA modifications in *Saccharomyces cerevisiae*. Our approach revealed several novel biosynthetic pathways for RNA modifications and led to the discovery of signature changes in the spectrum of tRNA modifications in the damage response to mechanistically different toxicants. This is illustrated with the RNA modifications Cm, m^5^C, and m^2^_2_G, which increase following hydrogen peroxide exposure but decrease or are unaffected by exposure to methylmethane sulfonate, arsenite, and hypochlorite. Cytotoxic hypersensitivity to hydrogen peroxide is conferred by loss of enzymes catalyzing the formation of Cm, m^5^C, and m^2^_2_G, which demonstrates that tRNA modifications are critical features of the cellular stress response. The results of our study support a general model of dynamic control of tRNA modifications in cellular response pathways and add to the growing repertoire of mechanisms controlling translational responses in cells.

## Author Summary

While the genetic code in DNA is read from four nucleobase structures, there are more than 100 ribonucleoside structures incorporated as post-transcriptional modifications mainly in tRNA and rRNA. These structures and their biosynthetic machinery are highly conserved, with 20–30 present in any one organism, yet the larger biological function of the modifications has eluded understanding. To this end, we developed a sensitive and precise mass spectrometric method to quantify 23 of the 25 ribonucleosides in the model eukaryotic yeast, *Saccharomyces cerevisiae*. We discovered that the spectrum of ribonucleosides shifts predictably when the cells are exposed to different toxic chemical stimulants, with these signature changes in the spectrum serving as part of the cellular survival response to these exposures. The method also revealed novel enzymatic pathways for the synthesis of several modified ribonucleosides. These results suggest a dynamic reprogramming of the tRNA and rRNA modifications during cellular responses to stimuli, with corresponding modifications working as part of a larger mechanism of translational control during the cellular stress response.

## Introduction

The complexity of the transfer RNA (tRNA) system confers great potential for its use in cellular regulatory programs. There are hundreds of tRNA-encoding genes in *S. cerevisiae* and human genomes, with extensive post-transcriptional processing that includes enzyme-mediated ribonucleoside modifications [1]. Considering both tRNA and ribosomal RNA (rRNA), there are more than 100 known ribonucleoside modifications across all organisms in addition to the canonical adenosine, guanosine, cytidine and uridine [2], [3]. In general, tRNA modifications enhance ribosome binding affinity, reduce misreading and modulate frame-shifting, all of which affect the rate and fidelity of translation [4]–[7]. However, information about the higher-level biological function of ribonucleoside modifications has only recently begun to emerge. We have approached this problem with a systems-level analysis of changes in the spectrum of ribonucleosides in tRNA as a function of cell stress, which has revealed novel insights into the biosynthesis of tRNA modifications and their role in cellular responses.

Emerging evidence points to a critical role for tRNA and rRNA modifications in cellular responses to stimuli, with evidence for a role in tRNA stability [8], [9], cellular stress responses [10]–[12] and cell growth [13]. We recently used high-throughput screens and targeted studies to show that the tRNA methyltransferase 9 (Trm9) modulates the toxicity of methylmethanesulfonate (MMS) in *S. cerevisiae* [11], [14]. This is similar to the observed role of Trm9 in modulating the toxicity of ionizing radiation [15] and of Trm4 in promoting viability after methylation damage [14], [16]. Trm9 catalyzes the methyl esterification of the uracil-based cm^5^U and cm^5^s^2^U to mcm^5^U and mcm^5^s^2^U, respectively, at the wobble bases of tRNA^UCU^-ARG and tRNA^CCU^-GLU, among others [17]. These wobble base modifications in the tRNA enhance binding of the anticodon with specific codons in mixed codon boxes [18]. Codon-specific reporter assays and genome-wide searches revealed that Trm9-catalyzed tRNA modifications enhanced the translation of AGA- and GAA-rich transcripts that functionally mapped to processes associated with protein synthesis, metabolism and stress signalling [11]. The resulting model proposes that specific codons will be more efficiently translated by anticodons containing the Trm9-modified nucleoside and that tRNA modifications can dynamically change in response to stress.

To assess the dynamic nature of tRNA modifications proposed by this model, we developed a systems-oriented approach using liquid chromatography-coupled, tandem quadrupole mass spectrometry (LC-MS/MS) to quantify the full set of tRNA modifications in an organism. Mass spectrometry-based methods have recently emerged as powerful tools for identifying and quantifying RNA modifications [19], [20]. We applied such an approach to quantify changes in the spectrum of tRNA modifications in yeast exposed to four mechanistically dissimilar toxicants. Multivariate statistical analysis of the data reveals dynamic shifts in the population of RNA modifications as part of the response to damage, with signature changes for each agent and dose. Further, analysis of yeast mutants lacking specific modification enzymes revealed novel biosynthetic pathways and compensatory or cooperative shifts in the levels of other modifications.

## Results/Discussion

### Development of an LC-MS/MS method to quantify modified ribonucleosides

**Figure 1:**
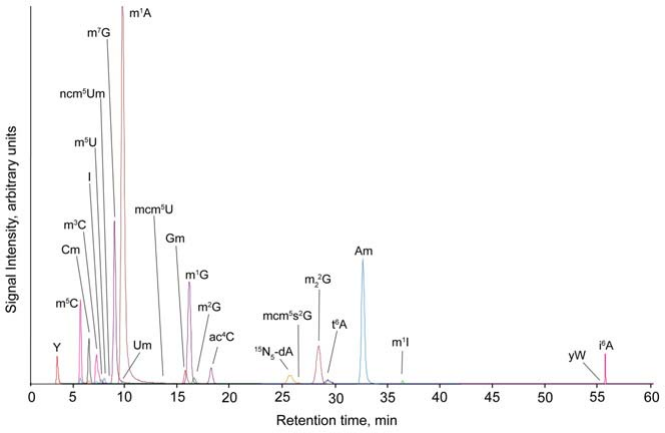
Total ion chromatogram from LC-MS/MS analysis of yeast tRNA ribonucleosides, as described in Materials and Methods.

As shown in Figure 1, we developed an LC-MS/MS method capable of quantifying 23 of the ∼25 known ribonucleoside modifications in cytoplasmic tRNA in *S. cerevisiae* [2], [3]. The method begins with isolation of small RNA species (<200 nt) and quantification of the tRNA content (∼80–90% of small RNA species). Individual ribonucleosides in enzymatic hydrolysates of tRNA were resolved by HPLC and identified by high mass accuracy mass spectrometry, by fragmentation patterns with collision-induced dissociation (CID) and by comparison to chemical standards. Each ribonucleoside was subsequently quantified by pre-determined molecular transitions during CID in the LC-MS/MS system. We were able to quantify 23 of the 25 tRNA modifications in yeast, with 2′-*O*-ribosyladenosine phosphate (Ar(p)) not detected in positive ion mode, possibly due to the negatively charged phosphate, and only tentative identification of ncm^5^Um by CID due to weak signal intensities.

A critical feature of our approach is quantitative rigor given the need for highly precise measurement of even small changes in the relative quantities of ribonucleosides. To this end, we used an Agilent Bioanalyzer (microfluidics-based sizing and quantification against an internal standard) for quantification of total tRNA species in the mixture of small RNA (85±5%, N = 39) and an internal standard ([^15^N_5_]-2′-deoxyriboadenosine) to minimize variation in the levels of the individual ribonucleosides. One caveat here is low-level contamination (a few percent) with 5S rRNA that also contains ribonucleoside modifications. We were able to obtain highly reproducible data for the signal intensity associated with each ribonucleoside (see Figure S1 for linearity of signal intensity for the 23 ribonucleosides). Multiple reaction monitoring (MRM) mode yielded no detectable background signal in the absence of tRNA hydrolysates except for i^6^A (9±2%). The method proved to be highly precise: 3±1% intra-day variance in average signal intensity and 12±10% inter-day variance in average fold-change values for each ribonucleoside in treated and untreated cells (294 analyses in three biological replicates over several weeks).

Analysis of tRNA from wild type cells revealed a three-log range of signal intensity, with I and ac^4^C producing the highest intensity and ncm^5^Um the lowest (Figure 1). In general, modifications can be categorized in high (I, ac^4^C, m^1^A, m^2^_2_G, Am, Y), medium (Cm, m^5^C, Gm, m^1^G, t^6^A, m^7^G, m^2^G, m^3^C, i^6^A) and low signal intensities (m^1^I, D, m^5^U, ncm^5^Um, mcm^5^U, mcm^5^s^2^U, Um, yW, ncm^5^U), with signal intensity reflecting both the abundance and mass spectrometric sensitivity for each ribonucleoside.

### Yeast exposure parameters

**Figure 2:**
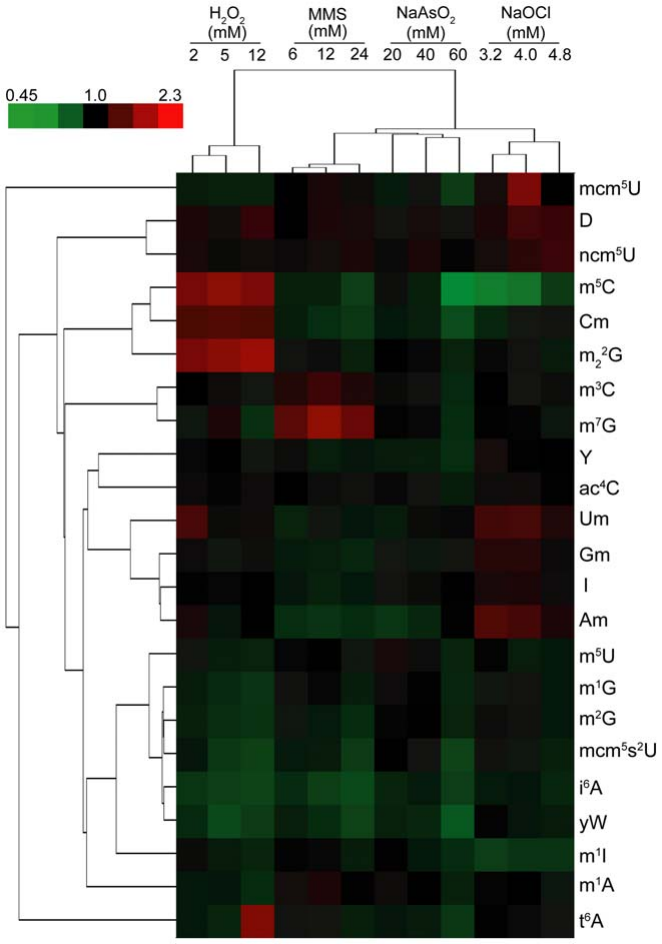
Hierarchical cluster analysis of toxicant-induced changes in tRNA modification spectra in wild-type yeast exposed to concentrations of MMS, H_2_O_2_, NaOCl, and NaAsO_2_ producing 20%, 50%, and 80% cytotoxicity. LC-MS/MS quantification of ribonucleosides was performed as described in Materials and Methods and data expressed as fold-change relative to unexposed controls (Table S2), with hierarchical cluster analysis performed on mean-centered data. The top-left color bar indicates the range of fold-change values.

To quantify the dynamics of tRNA modifications in cellular responses, we selected four well studied chemicals that possess distinct mechanisms of toxicity: MMS, hydrogen peroxide (H_2_O_2_), sodium arsenite (NaAsO_2_), and sodium hypochlorite (NaOCl, pK_a_ 7.5; ref. [21]). The behavior of yeast upon exposure to MMS, NaAsO_2_ and H_2_O_2_ has been extensively studied in terms of transcriptional response and cytotoxicity phenotyping [11], [22], [23]. We also chose NaOCl since it produces an oxidative stress distinct from that of H_2_O_2_ and could thus affect the tRNA modification spectrum differently. We then performed cytotoxicity dose-response studies in *S. cerevisiae* exposed to agents (Figure S2), choosing concentrations (Figure 2) that produced ∼20%, 50% and 80% cytotoxicity to ensure a common phenotypic endpoint for comparison.

One important issue with the methylating agent, MMS, was the possibility that changes in methyl-based modifications in tRNA could be due to both enzymatic methylation and direct chemical methylation. Literature precedent indicates that MMS reacts with DNA to form adducts mainly at guanine N^7^ (68%), adenine N^1^ (18%) and cytosine N^3^ (10%) [24], [25]. To address the extent of direct methylation of RNA by MMS, control studies were performed and revealed that direct alkylation by MMS contributes <25% to the cellular burden of m^7^G in small RNA, with the bulk of m^7^G arising by enzymatic methylation of tRNA (Figure S3). No other agent affected tRNA modifications in this manner, with changes in the relative quantities of the modifications resulting from alterations in biosynthesis, tRNA gene transcription or tRNA degradation.

### Reprogramming tRNA modifications during the stress response

With exposure and analytical parameters established, we tested the hypothesis that the spectrum of tRNA modifications would dynamically change as a function of the *S. cerevisiae* stress response. In addition, we predicted that these changes would serve as biomarkers of each exposure. Cells were exposed to three concentrations of each chemical and 23 tRNA modifications were quantified by LC-MS/MS, with the results shown in Tables S1 and S2, the latter as the ratio of treated to control signal intensities. A crude analysis of the data shows fold-changes ranging from 0.2 to 4, with 25% and 36% of the exposure data significantly different from control values by Student's t-test at p<0.05 and p<0.1, respectively (Table S2). These results point to the non-random and regulated nature of the exposure-induced changes in the levels of the tRNA modifications.

Multivariate statistical analyses revealed important patterns or signatures in the toxicant-induced changes in tRNA modifications. As shown in Figure 2, hierarchical clustering distinguished both agent- and dose-specific changes in the modification spectra, with unique patterns of increase and decrease apparent in all cases. H_2_O_2_ consistently increased the levels of m^5^C, Cm and m_2_^2^G and, at the highest concentration, t^6^A, with dose-dependent decreases in m^5^U, m^1^G, m^2^G, mcm^5^s^2^U, i^6^A, yW and m^1^A. MMS consistently increased the level of m^7^G, and decreased Am, m^5^C, Cm, mcm^5^s^2^U, i^6^A, and yW. NaAsO_2_ caused only decreases in modification levels at the highest concentration, most notably for mcm^5^U, m^3^C, m^7^G, mcm^5^s^2^U, i^6^A, yW, m^5^C, and Cm. Interestingly, the dose-response for NaOCl showed an inverse correlation between concentration and increased levels of Am and Um and decreased levels of m^5^C. Given the reproducibility of the data, the changes in tRNA modification spectra can be considered signature biomarkers of exposure for these four classes of chemical stressor.

**Figure 3:**
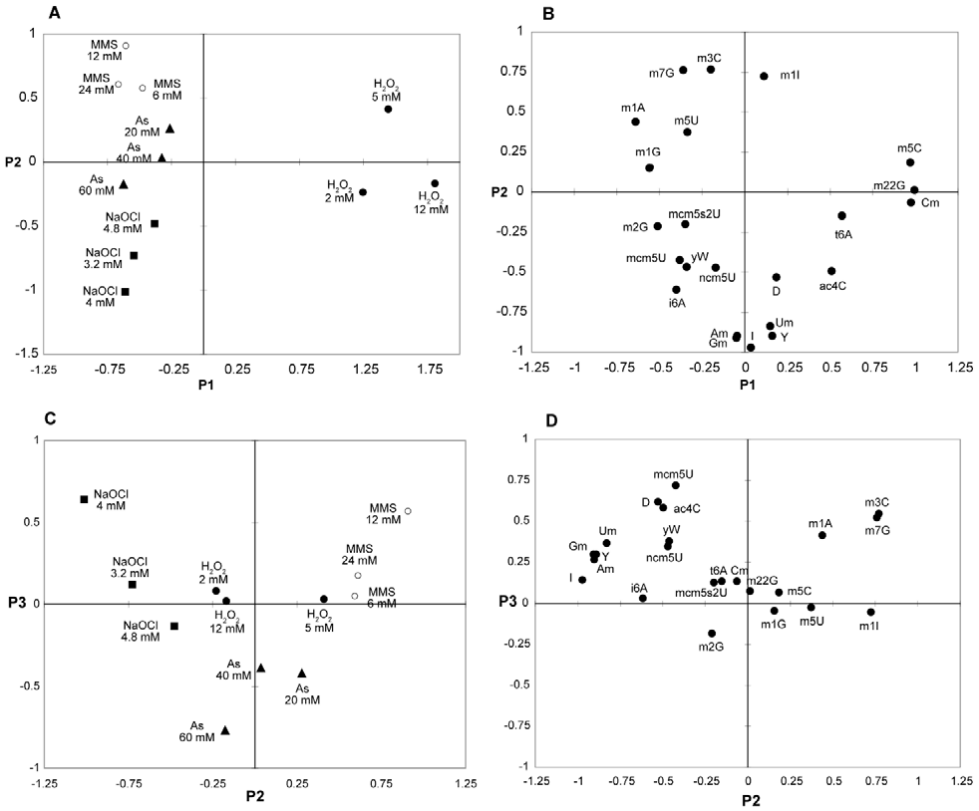
Principal component analysis (PCA) of changes in the levels of tRNA modifications caused by exposure to MMS, H_2_O_2_, NaOCl, and NaAsO_2_. Toxicant-induced changes in the relative quantities of 23 tRNA ribonucleoside modifications (Table S2) were subjected to PCA following mean centering and normalization of the fold-change data.

Principal component analysis (PCA) creates a model that reduces the complexity of a data set by identifying hidden correlations (the principal components) comprised of weighted, linear combinations of the original variables, with the first principal component (P1) accounting for the largest portion of the variation of the data and so on. The results of PCA of the dataset of nucleoside fold-change values (Table S2) are shown in Figure 3. With 88% of the variability expressed in the first 3 principal components (56%, 22% and 10%, respectively), individual agents contributed variance to each as shown in Table S3, with H_2_O_2_ contributing 74% in P1, MMS and NaOCl each contributing >40% in P2 and NaAsO_2_ contributing 53% in P3. The scores plots (Figure 3A, 3C) clearly distinguish the four agents, with H_2_O_2_-induced changes as the major determinant of P1 and with MMS, NaOCl and NaAsO_2_ distinguished best in P2. While H_2_O_2_ and NaOCl are negatively correlated in P1, they are more closely grouped in P2 and P3, which suggests that the changes in tRNA modifications reflect both common and unique facets of the toxic mechanism of each agent. For example, H_2_O_2_ and NaOCl are both oxidizing agents, but H_2_O_2_ generates hydroxyl radicals by Fenton chemistry while the protonated form of NaOCl yields hydroxyl radicals, chloramines and singlet oxygen [26]–[29]. Similarly, MMS and NaAsO_2_ are negatively correlated in P3 and more positively correlated in P2, with the latter consistent with recent evidence for alkylation-like adduction of arsenic to DNA and proteins following its metabolism [30], [31]. This would also explain the negative correlation of NaAsO_2_ and H_2_O_2_ in P1, while the recognized oxidative stress caused by arsenite [32] is consistent with a positive correlation between NaAsO_2_ and H_2_O_2_ in P2.

Both PCA (Figure 3B, 3D) and cluster analysis (Figure 2) revealed that m^5^C, m_2_^2^G, Cm and t^6^A are major features of the H_2_O_2_ response, while m^1^A, m^3^C and m^7^G were associated with MMS. Increases in Gm, Um, I and Am were responsible for the variance induced by NaOCl, which is consistent with the inversely related doses and levels for Am and Um observed in cluster analysis. NaAsO_2_ was poorly distinguished in P2, with only m^2^G accounting for variance only at the highest concentrations (Figure 2).

### tRNA modification biosynthetic pathways are critical to the stress response

**Figure 4:**
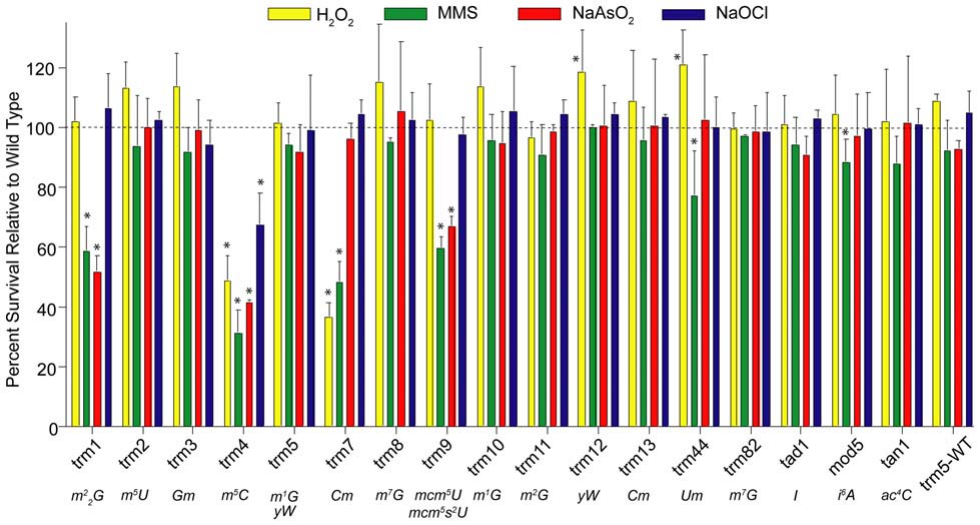
Phenotypic analysis of cytotoxicity induced by MMS, NaOCl, H_2_O_2_, and NaAsO_2_ in yeast mutants lacking *trm* tRNA methyltransferase and other modification genes. Data represent mean ± SD for three biological replicates. Asterisks denote values statistically different from unexposed controls by Student's t-test, p<0.05. Associated RNA modifications are listed below each enzyme.

The observation of toxicant- and dose-dependent changes in the levels of the 23 tRNA modifications is consistent with a model in which cells respond to toxicant exposure by modifying tRNA structure to enhance the synthesis of proteins critical to cell survival, as has been proposed in our earlier work with yeast exposure to MMS [11]. In this case, the conversion of cm^5^U to mcm^5^U by Trm9 was found to be critical for surviving MMS exposure [11]. To define the roles of specific tRNA modifications in the toxicant response, cytotoxicity phenotypic analyses were performed with yeast mutants lacking each of 13 *trm* tRNA methyltransferase genes and 3 other types of RNA modification biosynthetic genes. As shown in Figure 4, heightened sensitivity to H_2_O_2_ was observed in mutants lacking Trm4 and Trm7, which catalyze formation of two modifications elevated by H_2_O_2_ exposure: m^5^C and Cm, respectively [33], [34]. The simple explanation is that the increase in a specific tRNA modification is needed to promote an efficient stress response. However, m^2^_2_G was also elevated by H_2_O_2_ (Figure 2, Figure 3), yet loss of an enzyme involved in its biosynthesis, Trm1 [35], [36], did not confer H_2_O_2_ sensitivity (Figure 4). This behavior draws a comparison to mRNA, as it has been reported that many of the transcripts induced in response to a stress are not essential for viability during a challenge from that stress [37], [38]. MMS sensitivity was identified in *trm1*, *trm4* and *trm9* mutants, the latter as shown previously [11], whose corresponding proteins synthesize m^2^_2_G, m^5^C and mcm^5^U/mcm^5^s^2^U, respectively. However, these modifications were not strongly associated with MMS exposure in PCA (Figure 2, Figure 3). Somewhat surprisingly, loss of Trm1, Trm4, Trm7 and Trm9 conferred NaAsO_2_ sensitivity. These methyltransferases are responsible for m_2_^2^G, m^5^C, m^1^G (position 37) and mcm^5^u/mcm^5^s^2^U, respectively, of which only m^2^G was found to vary significantly in PCA (Figure 3). For NaOCl, only *trm4* was sensitive to exposure and the m^5^C product of Trm4 was not associated with NaOCl exposure (Figure 3). Again, this behavior parallels that of mRNA transcripts the levels of which do not change after exposure but that encode proteins important for viability after exposure [37], [38].

### Potential mechanisms linking tRNA modifications and the stress response

**Figure 5:**
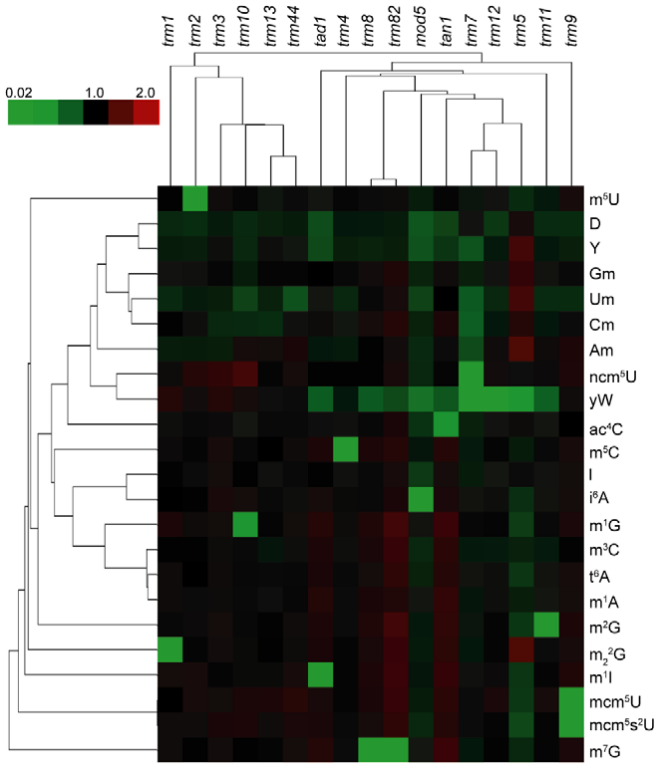
Cluster analysis visualization of changes in the relative levels of tRNA ribonucleoside modifications in mutants lacking ribonucleoside-modifying enzymes. The ratios of ribonucleoside levels from Table S5 were subjected to hierarchical cluster analysis. Red – increases; green – decreases. The top-left color bar indicates the range of fold-change values.

These results reveal a complex and dynamic control of tRNA modifications in cellular survival responses and suggest models for homeostasis of the modifications. One example involves modifications for which the biosynthetic mutant is sensitive to exposure but the modification level does not change in wild type cells following exposure (*e.g.*, MMS exposure and *trm1*/m^2^_2_G, *trm4*/m^5^C, *trm9*/mcm^5^U or mcm^5^s^2^U; Figure 2, Figure 3, Figure 4). The simplest explanation here is that the modification change occurs in a single tRNA species and the change is masked by an inverse change in the level of the modification in the larger population of tRNA molecules. As noted in Table S4, both m^2^_2_g and m^5^C occur in multiple tRNAs. A second explanation parallels the idea of both pre-existing mRNA and stressor-induced transcription during a stress response. We have observed stress-induced increases in the levels of several modifications required for the survival response (Figure 2, Figure 3; ref. [11]). However, other modifications may already exist on tRNA molecules involved in selective translation of stress response messages. In both cases, the modifications are absolutely required for survival, but some are already present in unstressed cells and others are induced. Finally, it is possible that a modification, though its level may not change, is required for the subsequent synthesis of other modifications that are critical to the survival response. Such “cooperativity” is suggested by data from *mod5*-deficient cells, in which i^6^A decreases by ∼75-fold while D is reduced by ∼2-fold. The presence of i^6^A may signal downstream biosynthetic events, with deficiencies promoting a general reprogramming of tRNA. Similarly, cells deficient in Trm82, a subunit of m^7^G methyltransferase, had a ∼7-fold reduction in m^7^G and a >1.5-fold increase in m^3^C, mcm^5^U, m^1^G, m^2^G, t^6^A, mcm^5^s^2^U and m_2_^2^G (Figure 5), which raises the possibility that Trm82 itself or m^7^G inhibits other tRNA modifying enzymes. With the caveat of possible increases in tRNA copy number, the ∼50% increase in these modifications suggests a pool of unmodified tRNA molecules, an observation supported by increases in m^3^C after exposure to MMS, mcm^5^U after exposure to NaOCl, and both t^6^A and m_2_^2^G after exposure to H_2_O_2_ (Figure 2, Figure 3).

Cooperativity could also explain the case in which the level of a modification changes significantly following exposure yet the mutant strain is not sensitive to the exposure. For example, loss of *trm1* did not confer sensitivity to H_2_O_2_ but its product, m^2^_2_G, rose significantly with H_2_O_2_ exposure (Figure 2, Figure 3, Figure 4). The stress-induced change in m^2^_2_G may be a response to a change occurring with another modification for which the mutant strain might be sensitive to the exposure. In support of this argument, m^5^C modifications increase along with m^2^_2_G after H_2_O_2_ exposure and deficiencies in the m^5^C-producing methyltransferase Trm4 confer sensitivity to H_2_O_2_. Wohlgamuth-Benedum *et al*. have also demonstrated such cooperativity among RNA modifications in their observation of the negative regulation of wobble position C-to-U editing by thiolation of a U at position 33 outside the anticodon in *T. brucei* [39].

Finally, there is the case in which a modification decreases with exposure to a stressor and a deficiency in the enzyme responsible for that modification confers sensitivity, as in the case of m^5^C, *trm4* and NaOCl (Figure 2, Figure 3, Figure 4). The population level of m^5^C may decrease with NaOCl exposure in spite of a protective increase in the level of m^5^C at some critical tRNA location. This may reflect a decrease in the transcription of tRNA substrates of Trm4 or the targeted degradation of specific tRNA species. It is important to note that biosynthetic redundancy, as in the case of Gm with Trm3 and Trm7, could mask any major changes in tRNA modification levels that are associated with mutational loss of one enzyme (Figure 5), yet loss of one of the redundant enzymes can induce sensitivity, such as the case of H_2_O_2_ and *trm7* (Figure 2, Figure 3, Figure 4). These observations lead to many questions that obviously require more mechanistic study to define the precise role of tRNA modifications in cellular responses to stress.

One consistent feature that arose from our studies of modifications affected by or protecting against toxicant exposure was the frequent involvement of the wobble position, 34 (Tables S4, S6). The correlation between the wobble modification and the importance of a corresponding enzyme after toxicant exposure is not surprising in light of recent observations of the critical role played by these modifications and anticodon loop ribonucleosides in translational fidelity and efficiency [4]. Controlled alteration of ribonucleoside structure at position 34, and that at the conserved purine at position 37, is proposed to allow reading of degenerate codons by modulating the structure of the anticodon domain to facilitate correct codon binding [4]. As the most frequently modified ribonucleosides, positions 34 and 37 also have the largest variety of modifications [40], [41], so it is reasonable that they would be extensively involved in translational control of the survival response. This is also consistent with our previous observation that mcm^5^U at the wobble position was critical to the translation of key protein synthesis and DNA damage response genes [11].

Perhaps more interesting is a potential role for putative non-anticodon loop ribonucleoside modifications in the survival response. For example, Trm44 is the 2′-*O*-methyltransferase in yeast responsible for formation of 2′-*O*-methyl-U (Um), which occurs only at position 44 in yeast tRNA [42], [43]. Loss of Trm44 conferred sensitivity to NaAsO_2_ exposure. This observation suggests three possibilities: (1) that Trm44 synthesizes or influences the synthesis of modifications at other positions in tRNA; (2) that Um occurs in positions other than 44 (*e.g*., anticodon loop); or (3) that Um(44) plays a role in modulating translation in response to NaAsO_2_ exposure. Another example involves Trm1 and m^2^_2_G at position 26. Current evidence suggests that m^2^_2_G occurs only at position 26 in yeast tRNA [43] and that Trm1 is the methyltransferase responsible for its formation [44]. The fact that loss of Trm1 conferred sensitivity to MMS and NaAsO_2_ exposure and that H_2_O_2_ exposure increased the level of m^2^_2_G again suggest the three possibilities analogous to those for Trm44 and Um. Similar arguments can be made for Trm3 and Gm at position 18 with NaOCl exposure, for Trm11 and m^2^G at position 10 with NaOCl and NaAsO_2_ exposure, and for Trm8/82 and m^7^G at position 46 with MMS exposure.

All of these observations point to participation of wobble and non-wobble RNA modifications in a complex and dynamic network of translational mechanisms in cellular responses. This expands the repertoire of translational control mechanisms, which includes recent discoveries about the effect of ribonucleoside modifications on tRNA stability [8], [9]. In this model, cell stress leads to rapid degradation of specific tRNAs and subsequent effects on translational efficiency. Another similar stress response involves cleavage of cytoplasmic transfer RNAs by ribonucleases released during the stress [10]. One consequence of these degradation pathways would be to decrease the amount of modified ribonucleoside detected in our assay, which may explain some of our observations with the toxicant stresses. Our approach to quantifying tRNA modifications provides information only about population-level changes, so the observed changes could result from modification of existing tRNA molecules or changes in the number of tRNA copies. Of particular importance here is the observation by Phizicky and coworkers that loss of m^7^G at position 46 leads to degradation of specific tRNAs [9], which suggests that our observation of changes in the levels of RNA modifications could be amplified by both reduction in the activity of modifying enzymes and by tRNA degradation. On the other hand, one argument against large increases in tRNA copy number arises from recent observations of repressed tRNA transcription during S-phase and, of direct relevance to the present studies, during replication stress induced by MMS, hydroxyurea and likely other toxicants [45]. Finally, our findings may also parallel recent work on tRNA charging. Reactive oxygen species have been implicated as a methionine misacylation trigger and modification status could help promote these programmed changes to the genetic code [12]. As we are beginning to appreciate the precision and coordinated nature by which cells mount a regulated stress-response, it is most likely the observed changes in tRNA modification levels promote multiple biological responses.

### Novel biosynthetic pathways for tRNA modifications

As recognized by several groups [19], [20], the LC-MS/MS platform facilitates definition of biosynthetic pathways for RNA modifications. This is illustrated in Table S5, which contains ratios of the basal levels of tRNA modifications in yeast mutants lacking various tRNA modification enzymes compared to wild type yeast, and in a heat map visual depiction of these ratios in Figure 5. These data corroborate known substrate/enzyme pairs [43] and further demonstrate the highly quantitative nature of our approach. For example, the level of m^1^I drops to nearly undetectable levels with loss of Tad1, the adenosine deaminase producing the inosine precursor to m^1^I [46]. That a diploid heterozygous mutant of *trm5*, the product of which catalyzes *N*-methylation of I [47], caused a ∼40% reduction in total m^1^I attests to the accuracy of our assay and demonstrate that gene dosage effects alter the level of tRNA modification. A similar ∼50% reduction in yW occurred in the *trm5* mutant due to the absence of the m^1^G(37) precursor to yW [47], while complete loss of Trm12, which methylates the 4-demethylwyosine precursor of yW, made yW undetectable. Other pathways critical to yW are apparent in the smaller decreases in yW (0.3– to 0.5-fold) occurred in cells deficient in other enzymes (Trm8, Trm82, Tad1, Mod5, Tan1, Trm11, Trm5; Figure 5, Table S5).

The data in Figure 5 also reveal several novel observations. Pintard *et al.* observed that Trm7 catalyzes 2′-*O*-methylation of G and C nucleosides at positions 32 and 34, but they could not detect the ncm^5^Um product of 2′-*O*-methylation of ncm^5^U [34]. While we could only tentatively identify ncm^5^Um, we observed a quantifiable signal for a species with the correct molecular transition for ncm^5^Um and observed that loss of Trm7 led to a lowering of putative ncm^5^Um to undetectable levels (Figure 5, Table S5). This supports their prediction that Trm7 catalyzes formation of ncm^5^Um in yeast.

Another example involves the formation of Um. While Trm44 catalyzes synthesis of Um at position 44 in tRNA(ser) [42], analysis of *trm* mutants in Figure 5 and Table S5 suggests a redundancy in methyltransferase activity capable of 2′-*O*-methylation of U(44), including Trm7, which methylates U at positions 32 and 34 [34], and Trm13 methylation of C and A at position 4 in several yeast tRNAs. Cells lacking Trm44, Trm7 or Trm13 have 53%, 50% and 76% of wild type levels of Um, respectively. More striking evidence for this redundancy arises in correlation analysis that revealed a strong covariance in the levels of tRNA modifications in cells lacking either Trm 44 or Trm 13 (Table S7; C = 0.87). This correlation ranks second highest in our analysis behind the two subunits of the m^7^G methyltransferase (Trm8 and Trm82; C = 0.95), which suggests possible functional redundancy for Trm44 and Trm13, with broader substrate specificities for either or both enzymes.

In summary, a quantitative bioanalytical approach to the study of tRNA modifications has revealed several novel biosynthetic pathways for RNA modifications and has led to the discovery of signature changes in the spectrum of tRNA modifications in the damage response to different toxicant exposures. The results support a general model of dynamic control of tRNA modifications in cellular response pathways and add to the growing repertoire of mechanisms controlling translational responses in cells [8]–[10], [13]. Further, these cellular response mechanisms almost certainly involve parallel changes in spectrum of ribonucleoside modifications in rRNA and perhaps other RNA species.

## Materials and Methods

### Materials

All chemicals and reagents were of the highest purity available and were used without further purification. 2′-*O*-Methyluridine (Um), pseudouridine (Y), N^1^-methyladenosine (m^1^A), N^2^,N^2^-dimethylguanosine (m^2^_2_G), and 2′-*O*-methylguanosine (Gm) were purchased from Berry and Associates (Dexter, MI). N^6^-Threonylcarbamoyladenosine (t^6^A) was purchased from Biolog (Bremen, Germany). N^6^-Isopentenyladenosine (i^6^A) was purchased from International Laboratory LLC (San Bruno, CA). 2′-*O*-Methyladenosine (Am), N^4^-acetylcytidine (ac^4^C), 5-methyluridine (m^5^U), inosine (I), 2-methylguanosine (m^2^G), N^7^-methylguanosine (m^7^G), 2′-*O*-methylcytidine (Cm), 3-methylcytidine (m^3^C), 5-methylcytidine (m^5^C), alkaline phosphatase, lyticase, RNase A, ammonium acetate, geneticine and desferrioxamine were purchased from Sigma Chemical Co. (St. Louis, MO). Nuclease P1 was purchased from Roche Diagnostic Corp. (Indianapolis, IN). Phosphodiesterase I was purchased from USB (Cleveland, OH). PureLink miRNA Isolation Kits were purchased from Invitrogen (Carlsbad, CA). Acetonitrile and HPLC-grade water were purchased from Mallinckrodt Baker (Phillipsburg, NJ). All strains of *S. cerevisiae* BY4741 were purchased from American Type Culture Collections (Manassas, VA).

### Exposure of *S. cerevisiae*

Cultures of *S. cerevisiae* BY4741 were grown to mid-log phase followed by addition of toxicants to the noted final concentrations (cytotoxicity of ∼20%, 50% and 80%): H_2_O_2_, 2, 5 or 12 mM; MMS, 6, 12 or 24 mM; NaAsO_2_, 20, 40 or 60 mM; NaOCl, 3.2, 4.0 or 4.8 mM. The sensitivity of the following mutant strains to toxicant exposure was also determined (doses producing ∼80% cytotoxicity in wild-type: 12 mM H_2_O_2_, 24 mM MMS, 60 mM NaAsO_2_, or 4.8 mM NaOCl): *trm1*, *trm2*, *trm3*, *trm4*, *trm7*, *trm8*, *trm9*, *trm10*, *trm11*, *trm12*, *trm13*, *trm44*, *trm82*, *tad1*, *mod5*, and *tan1*. Since *trm5* is essential, a diploid strain (*GBY1*) lacking one copy of *trm5* was used. After a 1 h, cells were collected and viability determined by plating.

### tRNA isolation

Following lyticase treatment (50 units) in the presence of deaminase inhibitors (5 µg/ml coformycin, 50 µg/ml tetrahydrouridine) and antioxidants (0.1 mM desferrioxamine, 0.1 mM butylated hydroxytoluene), tRNA-containing small RNA species were isolated (Invitrogen PureLink miRNA kit) and the tRNA quantified (Agilent Series 2100 Bioanalyzer).

### Quantification of cytoplasmic tRNA modifications

Following addition of deaminase inhibitors, antioxidants and [^15^N]_5_-2-deoxyadenosine internal standard (6 pmol), tRNA (6 µg) in 30 mM sodium acetate and 2 mM ZnCl_2_ (pH 6.8) was hydrolyzed with nuclease P1 (1 U) and RNase A (5 U) for 3 h at 37°C and dephosphorylated with alkaline phosphatase (10 U) and phosphodiesterase I (0.5 U) for 1 h at 37°C following addition of acetate buffer to 30 mM, pH 7.8. Proteins were removed by filtration (Microcon YM-10). Ribonucleosides were resolved with a Thermo Scientific Hypersil GOLD aQ reverse-phase column (150×2.1 mm, 3 µm particle size) eluted with the following gradient of acetonitrile in 8 mM ammonium acetate at a flow rate of 0.3 ml/min and 36°C: 0–18 min, 1–2%; 18–23 min, 2%; 23–28 min, 2–7%; 28–30 min, 7%; 30–31 min, 7–100%; 31–41 min, 100%. The HPLC column was coupled to an Agilent 6410 Triple Quadrupole LC/MS mass spectrometer with an electrospray ionization source where it was operated in positive ion mode with the following parameters for voltages and source gas: gas temperature, 350°C; gas flow, 10 l/min; nebulizer, 20 psi; and capillary voltage, 3500 V. The first and third quadrupoles (Q1 and Q3) were fixed to unit resolution and the modifications were quantified by pre-determined molecular transitions. Q1 was set to transmit the parent ribonucleoside ions and Q3 was set to monitor the deglycosylated product ions, except for Y for which the stable C-C glycosidic bond led to fragmentation of the ribose ring; we used the *m/z* 125 ion for quantification [48], [49]. The dwell time for each ribonucleoside was 200 ms. The retention time, *m/z* of the transmitted parent ion, *m/z* of the monitored product ion, fragmentor voltage, and collision energy of each modified nucleoside and ^15^N-labeled internal standard are as follow: D, 1.9 min, *m/z* 247→115, 80 V, 5 V; Y, 2.5 min, *m/z* 245→125, 80 V, 10 V; m^5^C, 3.3 min, *m/z* 258→126, 80 V, 8 V; Cm, 3.6 min, *m/z* 258→112, 80 V, 8 V; m^5^U, 4.2 min, *m/z* 259→127, 80 V, 7 V; ncm^5^U, 4.3 min, *m/z* 302→170, 90 V, 7 V; ac^4^C, 4.4 min, *m/z* 286→154, 80 V, 6 V; m^3^C, 4.4 min, *m/z* 258→126, 80 V, 8 V; ncm^5^Um, 5.5 min, *m/z* 316→170, 90 V, 7 V; Um, 5.1 min, *m/z* 259→113, 80 V, 7 V; m^7^G, 5.1 min, *m/z* 298→166, 90 V, 10 V; m^1^A, 5.7 min, *m/z* 282→150, 100 V, 16 V; mcm^5^U, 6.4 min, *m/z* 317→185, 90 V, 7 V; m^1^I, 7.3 min, *m/z* 283→151, 80 V, 10 V; Gm, 8.0 min, *m/z* 298→152, 80 V, 7 V; m^1^G, 8.3 min, *m/z* 298→166, 90 V, 10 V; m^2^G, 9.4 min, *m/z* 298→166, 90 V, 10 V; I, 10.9 min, *m/z* 269→137, 80 V, 10 V; mcm^5^s^2^U, 14.2 min, *m/z* 333→201, 90 V, 7 V; [^15^N]_5_-dA, 14.4 min, *m/z* 257→141, 90 V, 10 V; m^2^_2_G, 15.9 min, *m/z* 312→180, 100 V, 8 V; t^6^A, 17.2 min, *m/z* 413→281, 100 V, 8 V; Am, 19 min, *m/z* 282→136, 100 V, 15 V; yW, 34.2 min, *m/z* 509→377, 80 V, 5 V, and i^6^A, 34.4 min, *m/z* 336→204, 100 V, 17 V. The mass spectrometer monitored ions with the molecular transitions of D, Y, m^5^C, and Cm from 1 to 4 min; molecular transitions of m^5^U, ncm^5^U, ac^4^C, m^3^C, ncm^5^Um, Um, m^7^G, m^1^A, and mcm^5^U from 4 to 7 min; molecular transitions of m^1^I, Gm, m^1^G, and m^2^G from 7 to 10 min; molecular transitions of I, mcm^5^s^2^U, [^15^N]_5_-dA, m^2^_2_G, t^6^A, and Am from 10 to 30 min; molecular transitions of yW and i^6^A from 30 to 40 min. The identities of individual ribonucleosides were established by comparison to commercially available synthetic standards, high mass accuracy mass spectrometry, fragmentation patterns generated by collision-induced dissociation (CID) in a quadrupole time-of-flight mass spectrometer (QTOF) or MS^n^ analysis by ion trap mass spectrometry, with comparison to literature data (e.g., ref. [48]).

### Quantification of m^7^G in control and MMS-treated yeast

To assess the direct and indirect effects of MMS on levels of methylated ribonucleosides, the absolute levels of m^7^G were quantified in small RNA hydrolysates isolated from MMS-exposed and unexposed mutant and wild type strains of yeast by the LC-MS/MS method described above. Calibration curves were generated by mixing variable amounts of m^7^G (final concentrations of 0, 5, 50, 300, 600, 1000, and 2000 nM) with a fixed concentration of [^15^N]_5_-dA (40 nM). A volume of 10 µl of each solution was analyzed with the LC-MS/MS system described earlier.

### Statistical analysis of changes in the levels of tRNA modifications

Differences in the levels of ribonucleosides in exposed *versus* unexposed and in mutant *versus* wild-type yeast were analyzed by Student's t-test. Hierarchical clustering analyses were performed using Cluster 3.0. Data were transformed to log_2_ ratios of modification levels in treated cells relative to unexposed controls. Clustering was carried out using the centroid linkage algorithm based on the distance between each dataset measured using the Pearson correlation, with heat map representations produced using Java Treeview. Principal component analysis was performed using XLStat (Addinsoft SARL, Paris, France), with a Pearson correlation matrix consisting of data that were mean-centered and normalized to the standard deviation. Correlation analysis was used to assess the degree of covariance among the various sets of fold-change values for each mutant (Table S5), with correlation coefficients calculated using Excel (Microsoft).

## Supporting Information

Figure S1 Mass spectrometer signal intensities for tRNA ribonucleoside modifications. Small RNA isolates containing tRNA (85%) were enzymatically hydrolyzed and quantities ranging from 0.1 to 2 μg were analyzed by LC-MS/MS. Mass spectrometer signal intensities were determined for 23 of 25 modified ribonucleosides from yeast tRNA and plotted against total tRNA. Data represent mean ± SD for three analyses of the same sample.
(1.76 MB TIF)

Figure S2 Cytotoxicity dose-response studies with *S. cerevisiae* exposed to MMS, H_2_O_2_, NaAsO_2_ and NaOCl. Data represent mean ±SD for three biological replicates. The dotted line marks the 80% survival level.
(1.18 MB TIF)

Figure S3 Quantification of absolute level of m^7^G in different strains of yeast with or without MMS-exposure. Data represent mean ± SD for three biological replicates.
(2.88 MB TIF)

Table S1 Normalized mass spectrometer signal intensities for tRNA modifications in *S. cerevisiae* treated with four toxicants. Data represent mean ± SD for N = 3, with Student's t-test relative to control values.
(0.99 MB PDF)

Table S2 Fold-change values for *S. cerevisiae* tRNA modifications in treated cells relative to untreated controls. * Based on data from Table S1.
(0.07 MB PDF)

Table S3 Contribution of each agent to variance in principal component analysis.
(0.03 MB PDF)

Table S4 Relationships between conserved locations of tRNA ribonucleosides that are altered by exposure or that confer resistance to cytotoxicity in *S. cerevisiae*.
(0.09 MB PDF)

Table S5 Ratios of the levels of tRNA modifications in mutant strains relative to wild type *S. cerevisiae*. Underlined: Mutant was determined to be significantly different from wild type by Student's t-test with P<0.05; Yellow: ratios <0.02 (values of 0.00001 indicate undetectable ribonucleosides in the mutant strains); Green: ratios <0.6; Red: ratios >1.5.
(0.65 MB PDF)

Table S6 Locations of tRNA ribonucleosides affected by exposure to toxicants and critical to surviving toxicant exposure.
(0.04 MB PDF)

Table S7 Correlation coefficients between tRNA modification profiles for each mutant. Coefficients above 0.8 are shaded red and those between 0.5 and 0.8 are shaded pink.
(0.67 MB PDF)
