# Supplementary figures and images for: A Quantitative Systems Approach Reveals Dynamic Control of tRNA Modifications during Cellular Stress
Source: PLoS Genet. 2010 Dec 16;6(12):e1001247. doi: 10.1371/journal.pgen.1001247 (PMC3002981; doi:10.1371/journal.pgen.1001247)

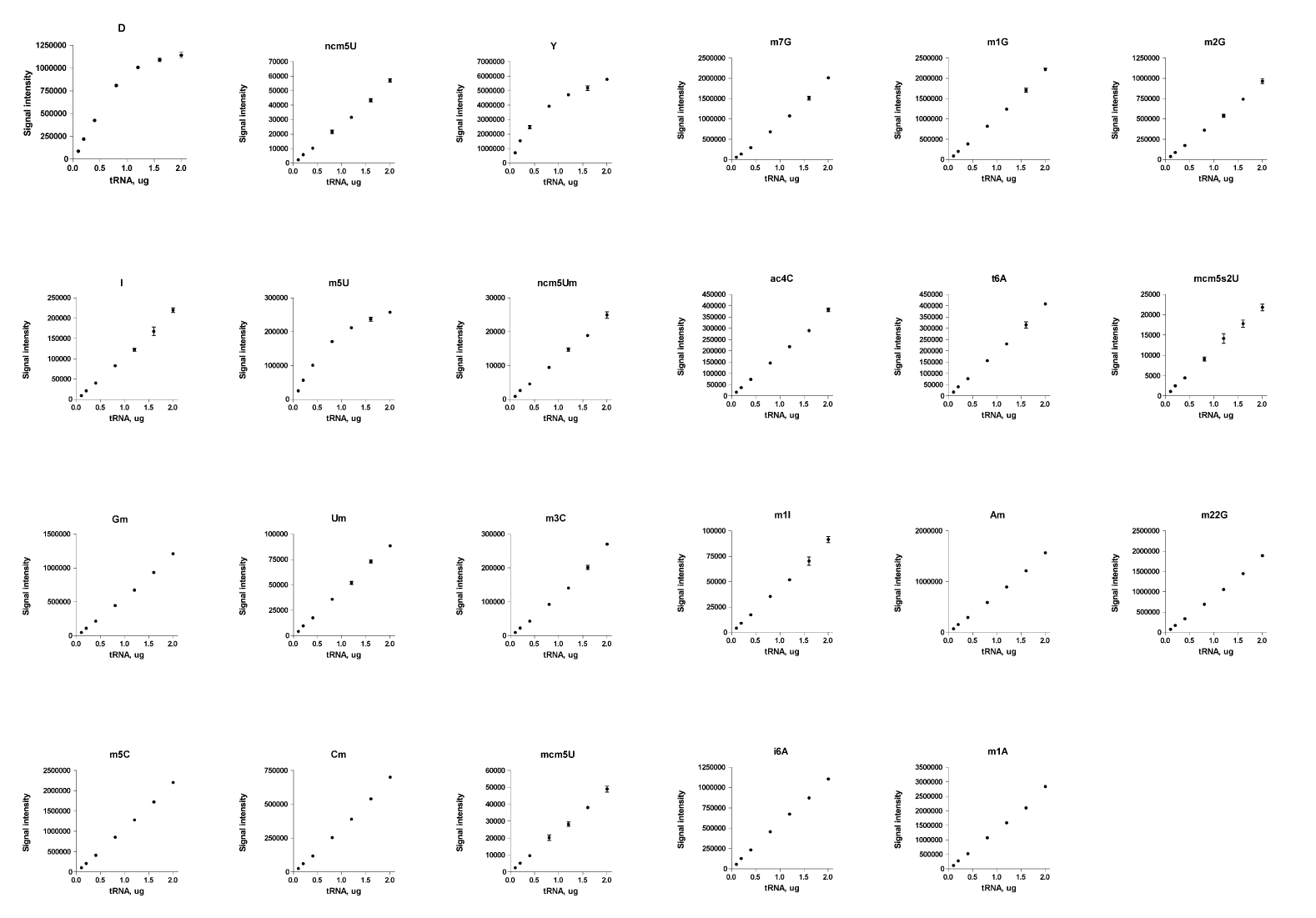

Supplement: Figure S1 — Mass spectrometer signal intensities for tRNA ribonucleoside modifications. Small RNA isolates containing tRNA (85%) were enzymatically hydrolyzed and quantities ranging from 0.1 to 2 μg were analyzed by LC-MS/MS. Mass spectrometer signal intensities were determined for 23 of 25 modified ribonucleosides from yeast tRNA and plotted against total tRNA. Data represent mean ± SD for three analyses of the same sample. (1.76 MB TIF) [file pgen.1001247.s001.tif]

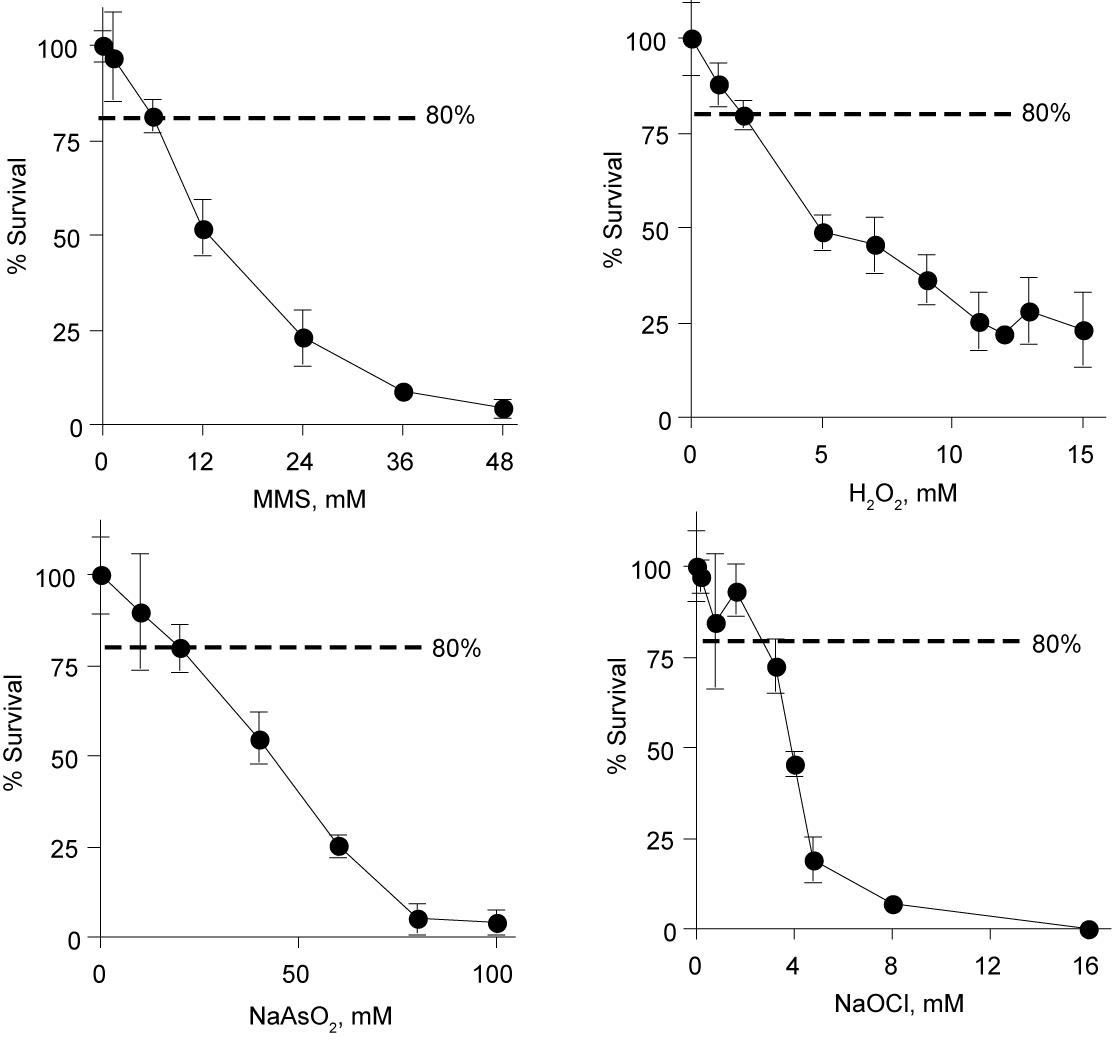

Supplement: Figure S2 — Cytotoxicity dose-response studies with S. cerevisiae exposed to MMS, H2O2, NaAsO2 and NaOCl. Data represent mean ±SD for three biological replicates. The dotted line marks the 80% survival level. (1.18 MB TIF) [file pgen.1001247.s002.tif]

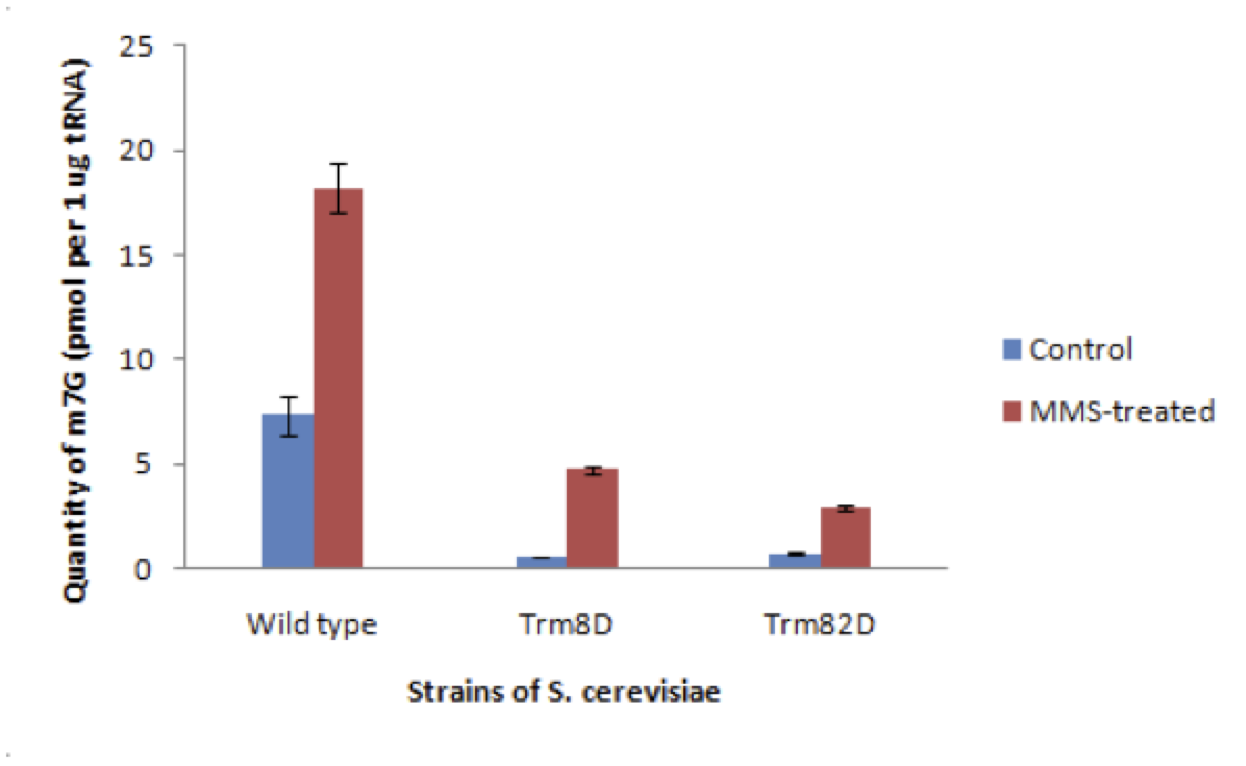

Supplement: Figure S3 — Quantification of absolute level of m7G in different strains of yeast with or without MMS-exposure. Data represent mean ± SD for three biological replicates. (2.88 MB TIF) [file pgen.1001247.s003.tif]
