# Supplementary material for: A Quantitative Systems Approach Reveals Dynamic Control of tRNA Modifications during Cellular Stress
Source: PLoS Genet. 2010 Dec 16;6(12):e1001247. doi: 10.1371/journal.pgen.1001247 (PMC3002981; doi:10.1371/journal.pgen.1001247)
Supplement: Table S1 — Normalized mass spectrometer signal intensities for tRNA modifications in S. cerevisiae treated with four toxicants. Data represent mean ± SD for N = 3, with Student's t-test relative to control values. (0.99 MB PDF) [file pgen.1001247.s004.pdf]

| | *Control* | | 2 mM $H_2O_2$ | | | 5 mM $H_2O_2$ | | | 12 mM $H_2O_2$ | | | 0.05% MMS | | | 0.1% MMS | | | 0.2% MMS | | |
|---|---|---|---|---|---|---|---|---|---|---|---|---|---|---|---|---|---|---|---|---|
| | *Average* | *SD* | *Average* | *SD* | *Ttest* | *Average* | *SD* | *Ttest* | *Average* | *SD* | *Ttest* | *Average* | *SD* | *Ttest* | *Average* | *SD* | *Ttest* | *Average* | *SD* | *Ttest* |
| **Y** | 82422 | 12427 | 86396 | 32354 | 0.85 | 83407 | 31689 | 0.96 | 74716 | 19789 | 0.60 | 77661 | 3308 | 0.56 | 70294 | 7321 | 0.22 | 73517 | 6950 | 0.34 |
| **D** | 1686 | 13 | 1986 | 281 | 0.14 | 1842 | 79 | 0.03 | 2254 | 171 | 0.00 | 1700 | 70 | 0.76 | 1985 | 221 | 0.08 | 1921 | 391 | 0.36 |
| **$ncm^5Um$** | 1664 | 135 | 1916 | 277 | 0.23 | 1759 | 214 | 0.55 | 1821 | 281 | 0.43 | 1769 | 179 | 0.46 | 1841 | 199 | 0.27 | 1941 | 135 | 0.07 |
| **I** | 250480 | 1392 | 257046 | 12547 | 0.42 | 241875 | 17587 | 0.45 | 250050 | 20166 | 0.97 | 223885 | 12822 | 0.02 | 210225 | 16080 | 0.01 | 220102 | 8971 | 0.00 |
| **$m^5U$** | 1675 | 88 | 1537 | 155 | 0.25 | 1430 | 221 | 0.15 | 1395 | 177 | 0.07 | 1619 | 133 | 0.58 | 1695 | 109 | 0.82 | 1518 | 128 | 0.16 |
| **Gm** | 45474 | 1577 | 49022 | 2453 | 0.10 | 41391 | 3670 | 0.15 | 42649 | 2117 | 0.14 | 39611 | 2775 | 0.03 | 38677 | 2047 | 0.01 | 36970 | 3531 | 0.02 |
| **Um** | 279 | 24 | 413 | 75 | 0.04 | 264 | 49 | 0.67 | 304 | 32 | 0.34 | 233 | 33 | 0.12 | 254 | 14 | 0.20 | 249 | 57 | 0.44 |
| **$m^5C$** | 39253 | 16194 | 75290 | 4063 | 0.02 | 84353 | 32490 | 0.10 | 77521 | 23005 | 0.08 | 33226 | 7583 | 0.59 | 33177 | 18704 | 0.69 | 28240 | 11893 | 0.40 |
| **$m^3C$** | 10889 | 703 | 10921 | 793 | 0.96 | 11637 | 4111 | 0.77 | 9960 | 230 | 0.10 | 13262 | 563 | 0.01 | 15328 | 412 | 0.00 | 13048 | 3166 | 0.31 |
| **Cm** | 25418 | 1842 | 38600 | 2591 | 0.00 | 39780 | 4380 | 0.01 | 38679 | 951 | 0.00 | 21883 | 1804 | 0.08 | 19956 | 2916 | 0.05 | 19031 | 1456 | 0.01 |
| **$mcm^5U$** | 245 | 22 | 213 | 26 | 0.17 | 207 | 65 | 0.39 | 207 | 21 | 0.09 | 248 | 11 | 0.89 | 284 | 33 | 0.17 | 265 | 32 | 0.43 |
| **$m^7G$** | 65612 | 9718 | 59284 | 7193 | 0.42 | 77334 | 45788 | 0.69 | 50903 | 1049 | 0.06 | 106987 | 6999 | 0.00 | 143214 | 7346 | 0.00 | 116659 | 49765 | 0.16 |
| **$m^1G$** | 59822 | 2872 | 51537 | 3469 | 0.03 | 48143 | 7020 | 0.06 | 45700 | 5714 | 0.02 | 55953 | 3142 | 0.19 | 57718 | 1657 | 0.33 | 51272 | 4363 | 0.05 |
| **$m^2G$** | 67775 | 3772 | 57313 | 6617 | 0.08 | 53298 | 11990 | 0.12 | 52085 | 12215 | 0.10 | 61161 | 7695 | 0.25 | 58984 | 3730 | 0.05 | 53313 | 5269 | 0.02 |
| **$ac^4C$** | 178172 | 8545 | 189350 | 12934 | 0.28 | 183742 | 5507 | 0.40 | 191576 | 16123 | 0.27 | 178450 | 7044 | 0.97 | 167917 | 18329 | 0.43 | 166783 | 15679 | 0.33 |
| **$t^6A$** | 60740 | 1623 | 53669 | 3863 | 0.04 | 49925 | 4521 | 0.02 | 123421 | 8103 | 0.00 | 55981 | 3326 | 0.09 | 55864 | 1870 | 0.03 | 51368 | 2652 | 0.01 |
| **$mcm^5s^2U$** | 513 | 65 | 458 | 54 | 0.32 | 382 | 13 | 0.03 | 367 | 35 | 0.03 | 448 | 16 | 0.17 | 444 | 50 | 0.22 | 376 | 35 | 0.03 |
| **$m^1I$** | 3128 | 317 | 2966 | 44 | 0.43 | 2702 | 417 | 0.23 | 2594 | 295 | 0.10 | 3054 | 41 | 0.71 | 3002 | 211 | 0.60 | 2675 | 348 | 0.17 |
| **Am** | 82676 | 2070 | 94388 | 5736 | 0.03 | 73858 | 12598 | 0.30 | 81631 | 5185 | 0.76 | 64714 | 6106 | 0.01 | 63191 | 2913 | 0.00 | 65214 | 12956 | 0.08 |
| **$m^2{}_2G$** | 133248 | 4375 | 257373 | 2009 | 0.00 | 280206 | 29780 | 0.00 | 309183 | 13971 | 0.00 | 122876 | 7381 | 0.10 | 125986 | 1482 | 0.05 | 112355 | 6407 | 0.01 |
| **$i^6A$** | 12673 | 3141 | 9592 | 1197 | 0.19 | 9094 | 1656 | 0.16 | 8926 | 1585 | 0.14 | 10061 | 849 | 0.24 | 9117 | 394 | 0.12 | 8627 | 433 | 0.09 |
| **yW** | 254 | 14 | 204 | 18 | 0.02 | 173 | 21 | 0.00 | 186 | 20 | 0.01 | 217 | 7 | 0.01 | 201 | 40 | 0.09 | 180 | 35 | 0.03 |
| **$m^1A$** | 141199 | 6656 | 123987 | 8584 | 0.05 | 125365 | 34747 | 0.48 | 111213 | 15492 | 0.04 | 155570 | 8122 | 0.08 | 166538 | 910 | 0.00 | 144187 | 24864 | 0.85 |

| | *Control* | | 20 mM $NaAsO_2$ | | | 40 mM $NaAsO_2$ | | | 60 mM $NaAsO_2$ | | | 3.2 mM NaOCl | | | 4.0 mM NaOCl | | | 4.8 mM NaOCl | | |
|---|---|---|---|---|---|---|---|---|---|---|---|---|---|---|---|---|---|---|---|---|
| | *Average* | *SD* | *Average* | *SD* | *Ttest* | *Average* | *SD* | *Ttest* | *Average* | *SD* | *Ttest* | *Average* | *SD* | *Ttest* | *Average* | *SD* | *Ttest* | *Average* | *SD* | *Ttest* |
| **Y** | 82422 | 12427 | 71278 | 6480 | 0.24 | 70560 | 13529 | 0.33 | 64565 | 10194 | 0.13 | 91794 | 13401 | 0.42 | 84461 | 8690 | 0.83 | 82114 | 13904 | 0.98 |
| **D** | 1686 | 13 | 646 | 129 | 0.79 | 780 | 152 | 0.68 | 643 | 153 | 0.78 | 821 | 165 | 0.55 | 986 | 90 | 0.17 | 954 | 168 | 0.25 |
| **$ncm^5Um$** | 1664 | 135 | 225 | 15 | 0.07 | 239 | 37 | 0.44 | 189 | 12 | 0.00 | 289 | 72 | 0.53 | 511 | 464 | 0.40 | 258 | 59 | 0.97 |
| **I** | 250480 | 1392 | 176791 | 24022 | 0.50 | 164125 | 15479 | 0.92 | 138153 | 4467 | 0.02 | 171485 | 8437 | 0.51 | 164512 | 5825 | 0.91 | 148475 | 14172 | 0.19 |
| **$m^5U$** | 1675 | 88 | 87325 | 11710 | 0.36 | 85861 | 5299 | 0.13 | 87900 | 7364 | 0.27 | 118264 | 16693 | 0.10 | 118582 | 3773 | 0.01 | 101952 | 9384 | 0.40 |
| **Gm** | 45474 | 1577 | 62376 | 8050 | 0.02 | 67489 | 1734 | 0.01 | 84669 | 5938 | 0.73 | 127864 | 8201 | 0.00 | 120941 | 1250 | 0.00 | 97826 | 7735 | 0.05 |
| **Um** | 279 | 24 | 242 | 39 | 0.15 | 235 | 31 | 0.07 | 182 | 11 | 0.00 | 280 | 16 | 0.63 | 259 | 3 | 0.09 | 250 | 45 | 0.25 |
| **$m^5C$** | 39253 | 16194 | 93100 | 11042 | 0.76 | 86918 | 8329 | 0.62 | 71430 | 3273 | 0.02 | 92691 | 5347 | 0.72 | 88108 | 4016 | 0.66 | 81446 | 9267 | 0.27 |
| **$m^3C$** | 10889 | 703 | 154839 | 21460 | 0.48 | 146691 | 14931 | 0.79 | 117558 | 9635 | 0.05 | 130656 | 16219 | 0.34 | 132287 | 3785 | 0.22 | 126490 | 14136 | 0.20 |
| **Cm** | 25418 | 1842 | 129941 | 21137 | 0.74 | 126325 | 13108 | 0.89 | 104067 | 7433 | 0.09 | 117919 | 11146 | 0.55 | 116662 | 2554 | 0.38 | 110416 | 12166 | 0.25 |
| **$mcm^5U$** | 245 | 22 | 203800 | 9591 | 0.24 | 209163 | 8493 | 0.39 | 217528 | 15976 | 0.85 | 252051 | 19311 | 0.11 | 260166 | 6120 | 0.02 | 237714 | 13057 | 0.26 |
| **$m^7G$** | 65612 | 9718 | 21142 | 2459 | 0.62 | 20440 | 1549 | 0.29 | 18978 | 1223 | 0.06 | 23781 | 3119 | 0.44 | 23964 | 775 | 0.15 | 21640 | 2214 | 0.81 |
| **$m^1G$** | 59822 | 2872 | 59705 | 5138 | 0.19 | 57821 | 6389 | 0.15 | 49945 | 3083 | 0.01 | 65494 | 6507 | 0.81 | 63792 | 4870 | 0.54 | 61653 | 8554 | 0.44 |
| **$m^2G$** | 67775 | 3772 | 4285 | 979 | 0.85 | 3672 | 587 | 0.23 | 3316 | 836 | 0.16 | 3018 | 326 | 0.01 | 3178 | 615 | 0.05 | 3186 | 242 | 0.00 |
| **$ac^4C$** | 178172 | 8545 | 522 | 69 | 0.93 | 487 | 69 | 0.55 | 374 | 32 | 0.04 | 492 | 40 | 0.53 | 478 | 58 | 0.43 | 447 | 65 | 0.25 |
| **$t^6A$** | 60740 | 1623 | 13327 | 2307 | 0.25 | 14062 | 2288 | 0.37 | 11717 | 1381 | 0.07 | 14069 | 604 | 0.28 | 14363 | 19 | 0.33 | 13277 | 1252 | 0.18 |
| **$mcm^5s^2U$** | 513 | 65 | 226 | 29 | 0.13 | 249 | 50 | 0.68 | 252 | 50 | 0.75 | 376 | 54 | 0.03 | 386 | 19 | 0.00 | 317 | 35 | 0.07 |
| **$m^1I$** | 3128 | 317 | 2513 | 461 | 0.33 | 2087 | 234 | 0.44 | 1827 | 302 | 0.10 | 2153 | 65 | 0.42 | 1885 | 109 | 0.02 | 1948 | 296 | 0.22 |
| **Am** | 82676 | 2070 | 48771 | 9709 | 0.73 | 44164 | 17102 | 0.55 | 24144 | 14414 | 0.06 | 27036 | 18513 | 0.12 | 28589 | 6125 | 0.04 | 38245 | 14717 | 0.28 |
| **$m^2{}_2G$** | 133248 | 4375 | 10785 | 1185 | 0.62 | 9524 | 1143 | 0.55 | 8215 | 677 | 0.09 | 10249 | 1516 | 0.98 | 9390 | 1575 | 0.54 | 9648 | 1818 | 0.69 |
| **$i^6A$** | 12673 | 3141 | 24804 | 4325 | 0.41 | 23893 | 4033 | 0.29 | 18889 | 7032 | 0.13 | 23094 | 11411 | 0.52 | 25705 | 3299 | 0.49 | 26144 | 5728 | 0.66 |
| **yW** | 254 | 14 | 164928 | 23674 | 0.89 | 155837 | 15424 | 0.56 | 135192 | 4577 | 0.01 | 156039 | 8431 | 0.44 | 150193 | 3051 | 0.12 | 140531 | 14810 | 0.10 |
| **$m^1A$** | 141199 | 6656 | 1012 | 191 | 0.76 | 1242 | 278 | 0.39 | 1096 | 302 | 0.87 | 1177 | 325 | 0.62 | 1339 | 175 | 0.13 | 1467 | 295 | 0.11 |
