# Supplementary material for: A Quantitative Systems Approach Reveals Dynamic Control of tRNA Modifications during Cellular Stress
Source: PLoS Genet. 2010 Dec 16;6(12):e1001247. doi: 10.1371/journal.pgen.1001247 (PMC3002981; doi:10.1371/journal.pgen.1001247)
Supplement: Table S2 — Fold-change values for S. cerevisiae tRNA modifications in treated cells relative to untreated controls. * Based on data from Table S1. (0.07 MB PDF) [file pgen.1001247.s005.pdf]

	H_2O_2, mM			MMS, mM			$NaAsO_2$, mM			NaOCl, mM		
Nucleoside	**2**	**5**	**12**	**6**	**12**	**24**	**20**	**40**	**60**	**3.2**	**4.0**	**4.8**
**D**	1.1776	1.0922	1.3363	1.0081	1.1772	1.1388	0.9256	1.1180	0.9220	1.1761	1.4134	1.3679
**Y**	1.0482	1.0119	0.9065	0.9422	0.8529	0.8920	0.8648	0.8561	0.7833	1.1137	1.0247	0.9963
**ncm5U**	1.1513	1.0570	1.0944	1.0630	1.1063	1.1663	0.9546	1.1713	1.0332	1.1096	1.2622	1.3835
**I**	1.0262	0.9656	0.9983	0.8938	0.8393	0.8787	0.9245	0.9488	0.9868	1.1434	1.1802	1.0783
**m5U**	0.9175	0.8539	0.8327	0.9667	1.0116	0.9063	1.1371	0.9442	0.8266	0.9740	0.8527	0.8812
**Gm**	1.0780	0.9102	0.9379	0.8711	0.8505	0.8130	0.9136	0.8983	0.9196	1.2373	1.2406	1.0666
**Um**	1.4809	0.9475	1.0909	0.8337	0.9114	0.8911	0.8604	0.9491	0.9600	1.4291	1.4703	1.2047
**m5C**	1.9181	2.1490	1.9749	0.8465	0.8452	0.7194	0.9374	0.8488	0.4640	0.5196	0.5495	0.7351
**m3C**	1.0029	1.0686	0.9147	1.2179	1.4076	1.1983	1.0562	0.9328	0.8045	1.0038	0.9196	0.9449
**Cm**	1.5186	1.5650	1.5217	0.8609	0.7851	0.7487	0.8823	0.8499	0.6719	0.8215	0.9144	0.9300
**mcm5U**	0.8664	0.8426	0.8425	1.0088	1.1558	1.0803	0.8685	0.9214	0.7289	1.1145	1.9679	0.9937
**m7G**	0.9036	1.1787	0.7758	1.6306	2.1827	1.7780	1.0280	0.9597	0.7887	1.0235	0.9729	0.8993
**m1G**	0.8615	0.8048	0.7639	0.9353	0.9648	0.8571	1.0790	1.0222	0.8192	0.9104	0.9218	0.8814
**m2G**	0.8456	0.7864	0.7685	0.9024	0.8703	0.7866	1.0417	1.0128	0.8343	0.9454	0.9353	0.8852
**ac4C**	1.0627	1.0313	1.0752	1.0016	0.9424	0.9361	0.9587	0.9269	0.8606	1.0784	1.0867	0.9813
**t6A**	0.8836	0.8219	2.0319	0.9217	0.9197	0.8457	0.8940	0.8658	0.7478	0.9807	0.9552	0.9232
**mcm5s2U**	0.8926	0.7445	0.7145	0.8738	0.8654	0.7323	0.9896	0.9235	0.7086	0.9320	0.9047	0.8471
**m1I**	0.9483	0.8638	0.8293	0.9763	0.9597	0.8553	1.0272	0.8801	0.7948	0.7234	0.7616	0.7636
**Am**	1.1417	0.8933	0.9874	0.7827	0.7643	0.7888	0.7512	0.8127	1.0196	1.5398	1.4564	1.1781
**m22G**	1.9315	2.1029	2.3204	0.9222	0.9455	0.8432	1.0138	0.9579	0.8310	0.9592	0.9232	0.8638
**i6A**	0.7569	0.7176	0.7043	0.7939	0.7194	0.6807	0.8232	0.8686	0.7238	0.8691	0.8873	0.8201
**yW**	0.8016	0.6805	0.7323	0.8534	0.7893	0.7091	0.8377	0.8156	0.6297	0.9715	0.8964	0.8647
**m1A**	0.8781	0.8879	0.7876	1.1018	1.1795	1.0212	1.0690	0.9924	0.8353	1.0369	0.9947	0.8978

* Shading color indicates significant difference from control by Student’s t-test: green, $p<0.05$; yellow, $p<0.1$
