# Supplementary material for: A Quantitative Systems Approach Reveals Dynamic Control of tRNA Modifications during Cellular Stress
Source: PLoS Genet. 2010 Dec 16;6(12):e1001247. doi: 10.1371/journal.pgen.1001247 (PMC3002981; doi:10.1371/journal.pgen.1001247)
Supplement: Table S3 — Contribution of each agent to variance in principal component analysis. (0.03 MB PDF) [file pgen.1001247.s006.pdf]

	**PC1**	**PC2**	**PC3**
**H_2O_2**	74%	6.9%	0.45%
**MMS**	11%	42%	21%
**$NaAsO_2$**	6.2%	2.8%	53%
**NaOCl**	8.7%	49%	26%
