# Supplementary material for: A Quantitative Systems Approach Reveals Dynamic Control of tRNA Modifications during Cellular Stress
Source: PLoS Genet. 2010 Dec 16;6(12):e1001247. doi: 10.1371/journal.pgen.1001247 (PMC3002981; doi:10.1371/journal.pgen.1001247)
Supplement: Table S4 — Relationships between conserved locations of tRNA ribonucleosides that are altered by exposure or that confer resistance to cytotoxicity in S. cerevisiae. (0.09 MB PDF) [file pgen.1001247.s007.pdf]

Nucleoside	Gene	Location[a] (Occurrence)	H_2O_2 Qty[b]	H_2O_2 Tox[b]	MMS Qty	MMS Tox	NaOCl Qty	NaOCl Tox	$NaAsO_2$ Qty	$NaAsO_2$ Tox
m^2_2G	*TRM1*	26 (18)	+			+				+
m^5U	*TRM2*	54 (29)								
Gm	*TRM3*	18 (9)					+			
	TRM7	***34***(1)		+			+			+
m^5C	*TRM4*	***34*** (1), 40 (1), 48 (15), 49 (10)	+	+		+		+		+
m^1A	*TRM6*	58			+					
	TRM61	58								
Cm	*TRM7*	32 (3), ***34*** (1)	+	+						+
	TRM13	4 (3)								
m^7G	*TRM8/ TRM82*	46			+					
mcm^5U	*TRM9*	***34***	+			+	+		+?	+
mcm^5s^2U	*TRM9*	***34***								+
m^1G	*TRM10*									
	TRM5	37								
m^2G	*TRM11*	10					+?		+?	
	TRM112	10								
yW	*TRM12*	37					+			
	TRM7?									
	TRM5									
ncm^5Um	***TRM7?***									
Um	*TRM44*	44								+
I	*TAD1*	37					+			
i^6A	*MOD5*	37					+			
ac^4C	*TAN1*	12								
m^3C	*?*	32			+					
Y	*PUS1*	27, 28, **34**, **35**								

[a] Location and enzyme data taken from refs. 1-4; numbers indicate tRNA nucleotide position, with the number of occurrences in yeast tRNA in parentheses; bold: anticodon (positions 34-36); underlined: positions within the anticodon loop (positions 31-39).

[b] "Qty": change in the level of the ribonucleoside caused by the agent; "Tox": loss of the secondary modification biosynthetic gene causes to sensitivity to the agent
