# Supplementary material for: A Quantitative Systems Approach Reveals Dynamic Control of tRNA Modifications during Cellular Stress
Source: PLoS Genet. 2010 Dec 16;6(12):e1001247. doi: 10.1371/journal.pgen.1001247 (PMC3002981; doi:10.1371/journal.pgen.1001247)
Supplement: Table S5 — Ratios of the levels of tRNA modifications in mutant strains relative to wild type S. cerevisiae. Underlined: Mutant was determined to be significantly different from wild type by Student's t-test with P<0.05; Yellow: ratios <0.02 (values of 0.00001 indicate undetectable ribonucleosides in the mutant strains); Green: ratios <0.6; Red: ratios >1.5. (0.65 MB PDF) [file pgen.1001247.s008.pdf]

	trm1	trm2	trm3	trm4	trm7	trm8	trm82	tad1	mod5	tan1	trm9	trm10	trm11	trm12	trm13	trm44	trm5
**D**	0.713842	0.6843	0.799161	0.828137	0.899072	0.825256	0.796538	0.544596	0.514717	0.583547	0.687507	0.706263	0.695729	0.630171	0.753443	0.793753	1.224693
**I**	1.026083	0.947245	1.184603	0.916817	0.796736	0.960603	1.223109	1.015979	0.624147	1.207896	1.164556	1.0172	0.892598	0.877719	0.909553	0.959943	0.948123
**m5U**	0.987038	0.00001	1.127997	0.971096	0.853232	0.945465	1.114468	0.879221	0.785055	1.037878	1.211402	0.972925	0.824946	0.893913	0.857149	0.95087	0.689327
**ncm5Um**	1.115053	1.442154	1.557688	1.00714	0.00001	0.99317	1.240788	1.010364	0.714159	0.97679	1.332745	1.804322	1.088102	1.150931	1.045787	1.204891	0.935474
**Gm**	0.892365	0.917907	0.969365	1.057554	0.765217	1.150425	1.355311	1.001871	0.743076	1.143667	0.95433	0.797357	0.907308	0.892193	1.034241	1.032041	1.583797
**Um**	0.707096	0.799301	0.772762	0.721069	0.498528	1.063909	1.225548	0.888201	0.582973	1.000561	0.68786	0.580268	0.696481	0.72486	0.755065	0.529322	1.798396
**m3C**	1.009635	1.007132	1.117877	0.925553	0.818491	1.274988	1.640635	1.30592	0.720901	1.474109	0.984104	0.964265	0.804095	0.805582	0.83824	0.931621	0.754647
**m5C**	1.082153	1.039257	1.226132	0.074026	0.805458	1.312151	1.443209	1.315717	0.838067	1.44199	1.210642	1.057321	0.965305	0.979524	0.993751	1.10052	0.791606
**Cm**	0.989427	0.929491	0.725017	0.870177	0.47594	1.201562	1.43464	1.120126	0.756758	1.305789	0.949605	0.709137	0.830921	0.832116	0.68439	0.911343	1.403351
**mcm5U**	1.00257	1.233468	1.156333	1.043397	0.945752	1.286914	1.639825	1.240346	0.788122	1.466071	0.00001	1.302712	1.20087	1.265654	1.309793	1.449138	0.627248
**m7G**	1.127881	1.013837	1.152332	0.947088	0.828513	0.114377	0.127359	1.4128	0.876977	1.677075	1.266986	1.018026	0.977665	1.02766	1.04834	1.173779	0.721473
**m1G**	1.307866	1.100389	1.156634	1.111559	1.055314	1.341812	1.757386	1.416961	0.904092	1.658933	1.260681	0.298146	0.960458	0.977426	0.974135	1.157466	0.607465
**m2G**	1.057559	1.092171	1.173847	0.951106	0.844212	1.358752	1.765857	1.316199	0.825633	1.522788	1.308046	1.058935	0.00001	0.973728	1.02583	1.12363	0.635986
**ac4C**	0.923116	0.957369	1.082035	0.909021	0.752637	1.061283	1.293255	0.939698	0.65876	0.30954	1.004896	0.874492	0.885059	0.891602	0.961265	0.965705	1.118684
**t6A**	1.097099	0.993229	1.106762	0.956773	0.90882	1.185038	1.532206	1.279125	0.756742	1.425328	1.187497	1.067276	0.906737	0.930285	0.953587	1.069266	0.63846
**mcm5s2U**	1.130653	1.127265	1.287195	0.953813	0.949808	1.187338	1.546039	1.277672	0.724113	1.400445	0.00001	1.311106	1.038589	1.041001	1.130092	1.255393	0.561531
**m1I**	1.171277	1.210978	1.043962	1.044721	0.919583	1.33084	1.680196	0.00001	0.83825	1.598951	1.354337	1.076146	1.040276	1.079506	1.073863	1.212763	0.638423
**Am**	0.783387	0.792853	0.772943	0.800324	0.545612	1.015545	1.15388	0.828891	0.699742	0.945327	1.320257	1.188623	1.097077	1.13754	1.156384	1.295224	1.959991
**m22G**	0.00001	1.042508	1.149887	0.978005	0.833386	1.252354	1.649986	1.292432	0.800677	1.493383	1.22816	1.073447	0.940472	0.978967	0.992541	1.112964	1.963598
**i6A**	1.00373	1.013344	1.249815	1.071445	0.904218	0.954777	1.281665	1.203227	0.012504	1.26141	1.182784	1.178433	0.903244	0.929682	0.95893	0.933019	0.681736
**yW**	1.408351	1.147706	1.406329	0.826683	0.00001	0.488963	0.559251	0.48456	0.424868	0.512475	1.127805	1.206756	0.473142	0.00001	0.935482	0.944868	0.279563
**m1A**	1.099058	0.944103	1.112871	0.944254	0.838408	1.289652	1.379047	1.389095	0.883242	1.571972	1.17681	0.963162	0.88898	0.921767	0.937037	1.049542	0.767246
**ncm5U**	0.708553	0.640167	0.724605	0.711749	0.810952	0.993275	0.805267	0.606002	0.822832	0.743067	0.847963	0.886971	0.718471	0.762053	0.930234	0.869326	0.91634
