# Supplementary material for: A Quantitative Systems Approach Reveals Dynamic Control of tRNA Modifications during Cellular Stress
Source: PLoS Genet. 2010 Dec 16;6(12):e1001247. doi: 10.1371/journal.pgen.1001247 (PMC3002981; doi:10.1371/journal.pgen.1001247)
Supplement: Table S6 — Locations of tRNA ribonucleosides affected by exposure to toxicants and critical to surviving toxicant exposure. (0.04 MB PDF) [file pgen.1001247.s009.pdf]

Position in tRNA	Nucleosides Affected by Exposure	Nucleosides Critical to Surviving Exposure
10	2	
18	1	
26	1	1
31		
32	2	1
33		
34	5	5
35	1	
36		
37	3	
38		
39		
44		1
46	1	
58		
