# Supplementary material for: A Quantitative Systems Approach Reveals Dynamic Control of tRNA Modifications during Cellular Stress
Source: PLoS Genet. 2010 Dec 16;6(12):e1001247. doi: 10.1371/journal.pgen.1001247 (PMC3002981; doi:10.1371/journal.pgen.1001247)
Supplement: Table S7 — Correlation coefficients between tRNA modification profiles for each mutant. Coefficients above 0.8 are shaded red and those between 0.5 and 0.8 are shaded pink. (0.67 MB PDF) [file pgen.1001247.s010.pdf]

	trm1	*trm2*	*trm3*	*trm4*	*trm7*	*trm8*	*trm82*	*tad1*	*mod5*	*tan1*	*trm9*	*trm10*	*trm11*	*trm12*	*trm13*	*trm44*	*trm5*
trm1																	
trm2	0.29																
trm3	0.44	0.52															
trm4	0.08	0.15	0.18														
trm7	-0.11	-0.20	-0.19	0.16													
trm8	-0.10	0.18	-0.05	0.02	0.38												
trm82	-0.03	0.31	0.13	0.19	0.38	0.95											
tad1	0.04	0.20	0.31	0.06	0.31	0.23	0.32										
mod5	0.02	0.03	-0.08	-0.06	0.26	0.33	0.26	0.24									
tan1	0.16	0.33	0.23	0.20	0.51	0.39	0.50	0.58	0.46								
trm9	0.08	-0.02	0.16	-0.01	-0.17	-0.08	-0.05	-0.03	0.08	0.13							
trm10	0.13	0.42	0.63	0.07	-0.35	-0.07	0.01	0.03	-0.10	0.04	0.00						
trm11	-0.04	0.16	0.08	0.09	0.14	0.06	0.09	0.08	0.13	0.20	-0.18	0.23					
trm12	-0.12	0.19	0.05	0.19	0.44	0.42	0.46	0.38	0.41	0.54	-0.05	0.26	0.55				
trm13	0.14	0.46	0.40	0.19	0.17	0.11	0.19	0.14	0.22	0.29	-0.18	0.55	0.40	0.56			
trm44	0.24	0.48	0.44	0.25	0.20	0.22	0.32	0.27	0.42	0.48	-0.03	0.54	0.45	0.63	0.87		
trm5	-0.77	-0.24	-0.60	-0.14	-0.19	0.01	-0.06	-0.14	-0.06	-0.28	0.00	-0.28	0.13	0.10	-0.20	-0.30	
